# 

**Published:** 1853-07

**Authors:** 


THIS VOLUME WILL BE COMPLETED IN EIGHT NUMBERS.

VOL. II. NEW SERIES. No. 3.

### THE NORTH-WESTERN

### MEDICAL AND SURGICAL

### JOURNAL:

EDITED BY

W. B. HERRICK, M.D.,

PROFESSOR OF ANATOMY AND PHYSIOLOGY IN RUSH MEDICAL COLLEGE; SURGEON TO THE U. S. MARINE HOSPITAL, ONE OF THE SURGEONS OF THE ILLINOIS GENERAL HOSPITAL, ETC.

AND

H. A. JOHNSON, A.M., M.D.

JULY, 1853.

CHICAGO:

BALLANTYNE & CO., PUBLISHERS & PRINTERS,

NEW POST-OFFICE BUILDINGS, COR. OF CLARK AND RANDOLPH-STS., OPPOSITE THE SHERMAN HOUSE.

1853.

VOL X. WHOLE SERIES.

